# Goal-directed cerebral hemodynamic strategy decreases the incidence of postoperative delirium in patients with intracranial hypertension in major abdominal surgery

**DOI:** 10.1186/cc14541

**Published:** 2015-03-16

**Authors:** I Zabolotskikh, N Trembach

**Affiliations:** 1 Kuban State Medical University, Krasnodar, Russia

## Introduction

Increased intracranial pressure (ICP) adversely affects anesthesia due to a disturbed cerebral blood flow. In older patients this disturbance may increase the incidence of postoperative delirium (POD) and may lead to a poor outcome [1]. The standard hemodynamic protocol involves maintaining the mean arterial blood pressure (MAP), but in patients with intracranial hypertension it may not be enough to maintain adequate cerebral perfusion. The purpose of this study was to evaluate the protocol of maintaining cerebral perfusion pressure (CPP) in the prevention of postoperative delirium in older patients in abdominal surgery.

## Methods

A total of 132 ASA 3 patients, undergoing major abdominal surgery (duration 5.2 (4.3 to 6.5) hours) with ICP >12 mmHg evaluated by a venous ophthalmodynamometry [2], were included in our research. Patients were randomized into two groups: MAP group, in which MAP was maintained above 70 mmHg or within 20% from baseline (*n *= 78); or CPP group, in which CPP was maintained above 60 mmHg or within 20% from baseline (*n *= 54). ICP, MAP and CPP were assessed every hour of anesthesia. Time of recovery of consciousness, incidence of POD and length of stay in the ICU and in the hospital were also evaluated.

## Results

Initial ICP was 14 ± 3 mmHg in the MAP group and 15 ± 2 mmHg in the CPP group. During the anesthesia it was stable without any significant change. Decreasing of MAP after induction of anesthesia was similar in two groups and it was stable during the anesthesia. The frequency of use of vasopressors and infusion rate was higher in the CPP group. Time of recovery of consciousness in the MAP group was higher (28 ± 7 minutes vs. 18 ± 5 minutes (*P *< 0.05)). The incidence of postoperative delirium was higher in the MAP group (18% vs. 11% in the CPP group (*P *< 0.05)). There were no significant differences between two groups in other complications. Total length of stay in the ICU and in the hospital was higher in the MAP group (6 ± 2 days vs. 4 ± 2 (*P *< 0.05) and 15 ± 3 days vs. 12 ± 2 in the N group (*P *< 0.05)).

## Conclusion

A goal-directed hemodynamic protocol of maintaining CPP can decrease the incidence of POD in older patients with intracranial hypertension after major abdominal surgery compared with a protocol of maintaining MAP.
